# Expanding the Progeroid Laminopathy Spectrum: Clinical Variability and Later‐Onset Phenotype in Homozygous LMNA c.1579C>T (p.Arg527Cys) Associated Mandibuloacral Dysplasia

**DOI:** 10.1155/crig/5579887

**Published:** 2026-09-19

**Authors:** Eszter Sara Arany, David Zocche, Jan Cobben

**Affiliations:** ^1^ Queen Charlotte’s and Chelsea Hospital, Imperial College Healthcare NHS Trust, London, UK, nhs.uk; ^2^ North West Thames Regional Genetics Service, Northwick Park & St Mark’s Hospitals, London, UK; ^3^ Department of Paediatrics, University of Oxford, Oxford, UK, ox.ac.uk

**Keywords:** distal acro-osteolysis, lipodystrophy, LMNA, mandibuloacral dysplasia, progeria

## Abstract

Homozygous LMNA c.1579C>T p.(Arg527Cys) has previously been reported in seven families and is associated with a progeroid phenotype intermediate between Hutchinson–Gilford progeria syndrome (HGPS) and mandibuloacral dysplasia type A (MAD‐A). Due to the limited number of reported cases, the complete phenotypic spectrum associated with this variant remains poorly understood. Here, we describe two Syrian siblings born to consanguineous parents, both homozygous for the LMNA p.Arg527Cys variant. Unlike previously reported cases, these siblings demonstrated a notably delayed onset of clinical symptoms. Both patients exhibited severe generalized lipodystrophy, pronounced progeroid facial dysmorphism, mandibular hypoplasia with severe micrognathia, and pan‐digital acro‐osteolysis. Our findings expand the recognized clinical spectrum associated with the homozygous LMNA p.Arg527Cys variant and illustrate intrafamilial variability in age of onset within an otherwise relatively homogeneous phenotype.

## 1. Introduction

Progeria syndromes are a group of rare genetic disorders characterized by accelerated aging‐like features in children. It is estimated that approximately 350–400 children are affected worldwide [1].

Among the progeria syndromes, the most well‐known is the dominantly inherited Hutchinson–Gilford progeria syndrome (HGPS), which presents with infantile‐onset profound growth failure, skin and skeletal abnormalities, loss of subcutaneous fat, and cardiovascular complications, often leading to death in adolescence due to atherosclerotic disease [2]. HGPS is most commonly caused by a de novo pathogenic variant in the LMNA gene, typically the recurrent c.1824C>T (p.Gly608Gly) variant [3]. The LMNA gene encodes two major protein isoforms: lamin A and lamin C, which are structural components of the nuclear lamina widely expressed in various tissues. The nuclear lamina provides essential mechanical support and regulates chromatin organization, gene expression, and nuclear stability [4]. The HGPS variant activates a cryptic splice site, leading to the production of a truncated progerin protein, which disrupts the nuclear architecture leading to nuclear instability, telomere truncation, defects in genetic repair, and consequently premature aging phenotype [3].

Recently, other LMNA‐related atypical progeroid laminopathies have also been discovered with the most prevalent being mandibuloacral dysplasia (MAD) type A. This condition is inherited in an autosomal recessive manner and typically shows a milder phenotype presenting with early childhood‐onset mandibular hypoplasia, acro‐osteolysis, clavicular hypoplasia, delayed closure of cranial sutures, growth retardation, and partial lipodystrophy (typically affecting limbs and buttocks) without cardiovascular involvement [5]. It has been suggested that biallelic LMNA variants cause progressive prelamin‐A accumulation and heterochromatin loss leading to similar nuclear instability as observed in HGPS [6, 7]; however, the exact mechanism is still subject to debate. The most common causative variant of MAD‐A is the missense c.1580G>A p.(Arg527His) variant [8].

Beyond LMNA, progeroid laminopathies are also linked to defects in genes involved in lamin A/C maturation and nuclear‐lamina integrity, notably ZMPSTE24 [9] and BANF1 [10], causing MAD type B and Nestor–Guillermo Progeria Syndrome, respectively. The clinical and molecular spectrum of MAD‐A and MAD‐B has been comprehensively reviewed by Cenni et al. [11].

Although HGPS and MAD‐A have traditionally been regarded as distinct entities, accumulating evidence suggests they lie on a phenotypic continuum. To date, only a limited number of reports have described individuals carrying the rare homozygous LMNA c.1579C>T (p.Arg527Cys) variant. These individuals exhibit an intermediate phenotype, combining progeroid features characteristic of HGPS with skeletal abnormalities typical of MAD‐A [12–18]. Here, we report two siblings homozygous for the LMNA c.1579C>T (p.Arg527Cys) variant, who exhibit this distinctive phenotypic overlap but with Patient 2 exhibiting a significantly later onset (age 36 months) compared to previously described cases. These cases broaden our understanding of the clinical spectrum associated with this ultrarare variant.

### 1.1. Patient 1

The proband is a 4‐year‐old female child born to consanguineous first‐cousin parents originally from Syria. Phenotypic features are described using Human Phenotype Ontology (HPO) nomenclature. She was first seen in clinic at 2 years of age due to growth delay [HPO: HP:0001510], failure to thrive [HPO: HP:0001508], low body weight [HPO: HP:0004325], and progeroid facial appearance [HPO: HP:0005328]. Parental concerns emerged at approximately 1 year of age, coinciding with growth concerns identified during routine health surveillance.

Family history: A sister of father has a son (IV‐1) who is said to have identical facial features as the proband (IV‐2), has short stature [HPO: HP:0004322], sparse scalp hair [HPO: HP:0002209], and low body weight [HPO: HP:0004325], but normal intelligence at the age of 17 years (no further information available). Parents had a normal phenotype.

The pregnancy was uncomplicated, resulting in a vaginal delivery at 38 weeks with a birth weight of 2.83 kg. Initial development during the first year was unremarkable, with normal growth trajectories.

At approximately 18 months, growth delay [HPO: HP:0001510] became apparent. By 4 years and 9 months, she had short stature [HPO: HP:0004322] and postnatal growth retardation [HPO: HP:0008897], with a height of 98 cm (1st centile); low body weight [HPO: HP:0004325], with a weight of 10.25 kg below the 0.4th centile (previously 2nd centile); and microcephaly [HPO: HP:0000252], with a head circumference of 47 cm (< 0.4th centile).

Physical examination revealed significant dysmorphic features, including a narrow face [HPO: HP:0000275], slight proptosis [HPO: HP:0000520] and ptosis [HPO: HP:0000508], mandibular hypoplasia, micrognathia [HPO: HP:0000347], a narrow nose [HPO: HP:0000460] with a narrow nasal ridge [HPO: HP:0000418], and a narrow mouth [HPO: HP:0000160] with limited opening. The patient exhibited thin skin [HPO: HP:0000963] of the face with prominent superficial veins [HPO: HP:0001015], white star‐shaped depigmentation of the skin at the inner eye corners, slight freckling [HPO: HP:0001480] of both cheeks, sparse scalp hair [HPO: HP:0002209], and diminished subcutaneous fat and decreased muscle mass [HPO: HP:0003199] throughout the body.

Nail dysplasia [HPO: HP:0002164] with thickened skin [HPO: HP:0001072] and joint swelling [HPO: HP:0001386] over the interphalangeal joints, affecting all fingers bilaterally, was present. X‐rays of both hands confirmed the presence of extensive acro‐osteolysis of distal phalanges [HPO: HP:0009699]. Joint stiffness [HPO: HP:0001387] and limitation of joint mobility [HPO: HP:0001376] at the knees impaired physical activity, necessitating significant assistance in daily activities such as dressing. She experienced generalized muscle weakness [HPO: HP:0003324], with exercise intolerance [HPO: HP:0003546] and fatigue [HPO: HP:0012378] after walking short distances (around 15 m) and had difficulty transitioning from lying to standing positions. Notably, she had sustained recurrent humeral fractures [HPO: HP:0002757] following minor falls on two occasions.

Developmental milestones, including fine motor skills, language, and social interaction, were age‐appropriate, with no intellectual deficits identified. Vision and hearing were unaffected, and she was successfully attending mainstream education, proficiently speaking Arabic and some English.

Biochemical investigations indicated low iron and vitamin D levels, which improved with supplementation. Imaging studies revealed bilateral acro‐osteolysis of fingers on hand radiographs, soft tissue swelling over distal interphalangeal joints, and abnormal rib morphology [HPO: HP:0000772] on chest x‐rays, with modeling deformities of the 2nd and 3rd ribs and normal clavicles. ECG was normal, and echocardiogram was not performed. Metabolic tests were not performed in the proband since these investigations are not first‐tier testing for dysmorphism with additional features in current UK pathways.

We performed a chromosomal microarray, which showed a normal female profile (arr[GRCh38] 46,XX, no pathogenic copy number variants detected). We analyzed the proband’s DNA by trio whole‐genome sequencing, applying the Genomics England PanelApp virtual panel R27 Pediatric Disorders (version 35.2). This analysis identified a homozygous LMNA c.1579C>T p.(Arg527Cys) variant, consistent with autosomal recessive MAD type A with lipodystrophy (MIM 248370). Both parents were confirmed as heterozygous carriers.

She continues to receive ongoing physical and occupational therapy to manage her symptoms.

### 1.2. Patient 2

The younger brother of the proband, born via uncomplicated vaginal delivery at term, initially exhibited no remarkable symptoms—he was seen by us when his sister was first diagnosed, and at that time, age 2 years, he exhibited no symptoms of the condition, with normal growth and facial features. At age 3 years, however, similar features to his sister became evident, including growth delay [HPO: HP:0001510], generalized muscle weakness [HPO: HP:0003324], and progeroid facial appearance [HPO: HP:0005328].

Physical examination revealed deeply set eyes [HPO: HP:0000490], marked micrognathia [HPO: HP:0000347], a narrow nose [HPO: HP:0000460], high palate [HPO: HP:0000218], sparse scalp hair [HPO: HP:0002209], and marked brachydactyly [HPO: HP:0001156] with broad fingertips [HPO: HP:0011300] and joint swelling [HPO: HP:0001386]. He displayed generalized lipodystrophy [HPO: HP:0009125], poor dentition, and lower limb muscle weakness [HPO: HP:0007340] but had a normal cardiovascular examination and no organomegaly. At evaluation, his height was 86 cm (< 0.4th centile) and his weight was 9.89 kg (< 0.4th centile), with low body weight [HPO: HP:0004325], significantly below average for his age.

Intellectual and social development remained unaffected. In view of his sister’s molecular diagnosis, targeted Sanger sequencing of LMNA was performed, which confirmed that he harbored the familial homozygous LMNA c.1579C>T p.(Arg527Cys) variant.

## 2. Discussion

Here, we present two patients homozygous for the LMNA c.1579C>T (p.Arg527Cys) variant who exhibited an overlap phenotype of HGPS and MAD‐A. Although the proband’s onset at approximately 18 months overlaps with the timing reported in Case IX, the younger brother’s presentation was later, with clinical features becoming apparent only at around 36 months of age (Table 1). Some of their features are typically observed in MAD‐A, including progressive thinning of scalp hair without complete alopecia, atrophic skin with hypopigmentation patches, mandibular hypoplasia, and acro‐osteolysis. However, the siblings also displayed marked growth retardation, prominent progeroid facial features, generalized lipodystrophy, and no evidence of clavicular hypoplasia more characteristic of HGPS. Although their clavicles appeared normal on current radiographs, clavicular hypoplasia and osteolysis are progressive features of progeroid laminopathies and may become apparent with age. Although not assessed in our patients, prelamin A could be examined in fibroblasts or exfoliated buccal mucosal cells; notably, previous analysis of homozygous LMNA p.Arg527Cys fibroblasts demonstrated marked nuclear morphological abnormalities [12].

**TABLE 1:** Comparison of phenotypic features in patients with homozygous LMNA c.1579C>T variant.

Features	Case I [12]	Case II [13]	Case III [13]	Case IV [14]	Case V [14]	Case VI [15]	Case VII [15]	Case VIII [15]	Case IX [16]	Case X [17]	Case XI [18]	Case XII	Case XIII	Total
Abnormality of the cardiovascular system	−	+	−	+	−	−	−	−	−	−	+	−	−	3/13
Abnormally high‐pitched voice	+	−	−	−	−	−	−	−	+	−	−	−	−	2/13
Alopecia	+	+^∗^	+^∗^	+	+^∗^	+^∗^	+	+^∗^	+	+^∗^	+^∗^	+	+	13/13
Aplasia/hypoplasia of the clavicles	+	+	+	+	+	−	−	−	−	+	−	−	−	6/13
Constipation	−	+	+	−	−	+	−	−	+	−	−	−	−	4/13
Convex nasal ridge	+	+	+	+	+	+	+	−	+	+	+	+	+	12/13
Delayed cranial suture closure	+	+	−	+	+^∗^	−	−	−	−	−	−	−	−	4/13
Dental crowding	+	+	−	+	−	+	+	−	+	+	−	−	+	8/13
Difficulty in swallowing	+	−	−	−	−	+	+	−	−	−	+	−	−	4/13
Failure to thrive	+^∗^	+	+^∗^	+	+	+^∗^	+	+	+	+	+	+^∗^	+^∗^	13/13
Gait disturbance	+	−	−	−	−	+	+	−	+	+	−	+	−	6/13
Hyperkeratosis	−	−	−	+^∗^	−	+^∗^	+	+	+	+	+^∗^	+	−	8/13
Hyperpigmentation of the skin	+	+	+	+	+	+	+	+	−	+	+	+	+	12/13
Hypopigmentation of the skin	−	−	−	−	−	−	−	−	−	+^∗^	+	+	+	4/13
Joint stiffness	+	+	+	+^∗^	+	+^∗^	+^∗^	+^∗^	+	+	+^∗^	+	−	12/13
Lipodystrophy (generalized)	−	+^∗^	−	+	+	−	−	−	−	+	+	+	+	7/13
Lipodystrophy (partial)	+	−	−	−	−	+	+	+	+	−	−	−	−	5/13
Micrognathia	+	+	+	+	+	+	+	+	+	+	+	+	+	13/13
Multiple skeletal anomalies	−	+^∗^	+	+	−	−	+	−	−	+	−	+	−	6/13
Obstructive sleep apnea	+	−	−	−	−	−	−	−	−	−	−	−	−	1/13
Osteolytic defects of the phalanges of the hand	+	+	+	+	−	+	+	+	+^∗^	+	+	+	+	12/13
Osteoporosis	−	−	−	+	−	−	−	−	−	+	−	−	−	2/13
Prominent forehead	+	+	+	+	+	+	+	+	+	+	+	+	+	13/13
Pruritus	−	+	+	−	−	−	−	−	−	−	−	−	−	2/13
Short stature	+	+	+^∗^	+	+	−	−	−	+	+	−	+	+	9/13
Thin skin	+	+	+	+	+	+	+	+	+	+	+	+	+^∗^	13/13
Age of onset (months)	12	2	4	5	6	10	12	8	18	4	6	18	36	

*Note:* The asterisk marks the patients’ presenting complaint.

The phenotype also shows substantial overlap with MAD‐B, caused by biallelic ZMPSTE24 variants, particularly through the generalized lipodystrophy, severe growth impairment, progeroid features, and acro‐osteolysis [19].

To date, seven families have been reported with the homozygous LMNA c.1579C>T (p.Arg527Cys) variant, all demonstrating a phenotype of overlapping features of both HGPS and MAD‐A. Typically, disease onset in these patients occurred within the first year of life, most commonly presenting with failure to thrive, alopecia, and joint stiffness. In contrast, our patients displayed a comparatively later disease onset, with failure to thrive manifesting in the proband at 18 months and progeroid features emerging in the younger sibling at 3 years of age. Both siblings exhibited significant generalized lipodystrophy. Furthermore, the proband experienced recurrent fractures following minor trauma, which is distinct from the predominantly distal osteolytic skeletal changes commonly described in other cases. Table 1 shows a detailed comparison of the phenotypes observed in previous cases and the present siblings. Skeletal involvement is a recognized feature of progeroid laminopathies, particularly in the form of mandibular and clavicular hypoplasia, acro‐osteolysis, osteopenia, or osteoporosis. However, recurrent long‐bone fractures are not emphasized in classic MAD type A. The humeral fractures observed in Patient 1 following minor trauma may reflect relevant skeletal fragility within the LMNA‐related progeroid spectrum.

The difference in apparent age of onset between the two siblings, despite the same homozygous LMNA variant, also highlights potential intrafamilial variability in this condition. This may reflect the influence of modifying genetic, epigenetic, developmental, or environmental factors, although the basis for this variability remains uncertain.

The two cases described here broaden the recognized phenotypic spectrum associated with the homozygous LMNA c.1579C>T (p.Arg527Cys) variant. In apparently healthy siblings of affected children, one should still be aware of later onset of features after the age of two and perform the appropriate genetic testing before excluding the diagnosis on clinical grounds alone.

## Author Contributions

Eszter Sara Arany: investigation, literature review, visualization, writing–original draft, and writing–review and editing. David Zocche: investigation, visualization, and writing–review and editing. Jan Cobben: conceptualization, clinical assessment of proband and brother, investigation, writing–review and editing, and supervision.

## Funding

No funding was received for this manuscript.

## Consent

Informed consent to publish clinical details and photographs was obtained from the patient’s parents.

## Conflicts of Interest

The authors declare no conflicts of interest.

## Data Availability

The data that support the findings of this study are available on request from the corresponding author.
